# Hygienic Treatment of Pulmonary Consumption

**Published:** 1858-04

**Authors:** 


### HYGIENIC TREATMENT OF PULMONARY CONSUMPTION.

The January number of the *American Quarterly Journal,* contains a paper by Dr. Flint, of Buffalo, on the "Treatment of Pulmonary Consumption by Hygienic Measures." Dr. Flint narrates the particulars of twenty-four cases of consumption, in all of which the old antiphlogistic methods, bleeding, emetics, low diet, confinement indoors, and such like injurious ingeniousnesses, were entirely avoided; but in which the tendency of the disease to natural arrest was favoured with the most happy results, by out door rough exercise and nutritious diet. Dr. Flint gathers from his observations in the treatment of this disease, that the measures are hygienic rather than medicinal. "The hygienic measures which are most important, are laborious exercise in the open air, conjoined with agreeable mental occupation; and, as conducive thereto, frequent change of business; the selection of an eligible climate and travelling are desirable, if not necessary; generous diet; and in many, if not most instances, the free use of alcoholic stimulants. The medicinal remedies, in addition to those which are simply palliative, are chiefly those of the tonic class."

It is most gratifying to see that the views so often expressed in this country of late, are thus independently making their way on the other side the Atlantic.

